# Lung function in very low birth weight infants after pharmacological and surgical treatment of patent ductus arteriosus - a retrospective analysis

**DOI:** 10.1186/s12887-016-0762-z

**Published:** 2017-01-06

**Authors:** Payman Barikbin, Hannes Sallmon, Silke Wilitzki, Joachim Photiadis, Christoph Bührer, Petra Koehne, Gerd Schmalisch

**Affiliations:** 1 Department of Neonatology, Charité University Medical Centre, Charitéplatz 1, 10117 Berlin, Germany; 2 Department of Congenital Heart Surgery, German Heart Institute, Augustenburger Platz 1, 13353 Berlin, Germany

**Keywords:** Patent ductus arteriosus, Ligation, Lung function test, Body plethysmography, Tidal breathing, Respiratory mechanics, Lung volume, VLBW infants

## Abstract

**Background:**

The indications and strategies for treatment of patent ductus arteriosus (PDA) are controversial, and the safety and long-term benefits of surgical PDA closure remain uncertain. The aim of this study was to compare the lung function of very low birth weight (VLBW) infants after successful PDA treatment with a cyclooxygenase inhibitor or secondary surgical ligation.

**Methods:**

A total of 114 VLBW infants (birth weight < 1500 g), including 94 infants (82%) with a birth weight < 1000 g, who received treatment for hemodynamically significant PDA (hsPDA), were examined at a median postmenstrual age of 48 weeks. All infants were initially given pharmacological treatment, and 40 infants (35%) required PDA ligation. Lung function testing (LFT) included tidal breathing measurements, measurement of respiratory mechanics assessed by the occlusion test, whole-body plethysmography, SF_6_ multiple breath washout, forced expiratory flow (V’max_FRC_) by the rapid thoracoabdominal compression technique, exhaled NO (FeNO), and arterialized capillary blood gas analysis.

**Results:**

On the day of the LFT, the 2 groups had similar postconceptional age and body weight. However, the PDA ligation group was more immature at birth (*p* < 0.001) and had reduced respiratory compliance (*p* < 0.001), lower V’max_FRC_ (*p* = 0.006), increased airway resistance (R_aw_) (*p* < 0.001), and impaired blood gases (*p* < 0.001). Multivariate analysis showed that PDA surgery was an independent risk factor for increased R_aw_.

**Conclusion:**

PDA ligation after failed pharmacological treatment is associated with impaired lung function as compared to successful pharmacological closure in infants at a postmenstrual age of 48 weeks. However, only Raw was independently affected by PDA ligation, while all other differences were merely explained by patient characteristics.

## Background

A persistently patent ductus arteriosus (PDA) affects about one-third of premature infants with very low birth weight (VLBW) and two-thirds of those with extremely low birth weight (ELBW). Left-to-right shunting through the PDA impairs systemic perfusion, and pulmonary overcirculation may lead to pulmonary edema, increased duration and intensity of mechanical ventilation, impaired alveolar development, and ultimately bronchopulmonary dysplasia (BPD) [1].

This provides the rationale for PDA treatment, although there are different opinions regarding the indications and treatment strategies based on consideration of the potential benefits and adverse effects [1, 2]. Pharmacological treatment with a cyclooxygenase (COX) inhibitor such as indomethacin or ibuprofen leads to PDA closure with a similar efficacy reported for both drugs [1, 3]. When medical treatment is contraindicated or unsuccessful, PDA ligation provides definitive closure. Although the morbidity and mortality rates following PDA ligation are generally low, previous studies have linked this procedure with severe complications such as left ventricular and respiratory compromise, pneumothorax, chylothorax, increased risk of BPD, thoracic scoliosis, and neurosensory impairment [4, 5]. However, it is unclear whether these adverse outcomes are causally associated with surgery itself or merely reflect the more severe illness and/or longer exposure to PDA-related circulatory effects in these patients [6]. Another potentially underestimated complication of PDA surgery is injury to the recurrent laryngeal nerve. This condition may result in voicing disorders and impediments to gas flow in the proximal airways with possible persistence of symptoms into adulthood [7].

The unfavorable short-term effects of PDA on pulmonary mechanics, formation of pulmonary edema, and higher ventilator dependence are well established. However, a direct role of PDA in the development of chronic lung disease/BPD has not yet been proven unequivocally. Several studies have assessed the short-term effects of pharmacological or surgical PDA closure on pulmonary function in animal models [8, 9] and in ventilated infants [10–12]. We hypothesize that different treatment effects can also be seen in VLBW infants by postnatal lung function testing (LFT). To our knowledge, no previous studies have compared LFT results of spontaneously breathing infants after pharmacological PDA treatment vs. secondary surgical closure during early infancy. The aim of this study was to compare LFT results of infants with PDA who achieved closure following successful pharmacological or secondary surgical treatment after failed pharmacological treatment.

## Methods

### Subjects

This retrospective study examined 114 VLBW infants (birth weight < 1500 g) who had hemodynamically significant PDA (hsPDA) and underwent LFT during early infancy. All patients initially received pharmacological treatment; this initial treatment failed in 40 patients, who then underwent PDA ligation. We used the clinical risk index for babies (CRIB) score to assess initial neonatal risk of hospital mortality. This scoring system considers birth weight, gestational age, maximum and minimum fraction of inspired oxygen, maximum base excess during the first 12 h of life, and presence of congenital malformations. Infants were assessed between November 2004 and February 2014 at a median postmenstrual age of 48 weeks. LFT is a part of our routine follow-up care of infants after neonatal intensive care. Infants with congenital heart disease, neuromuscular disease, or thoracic wall deformities were excluded.

An hsPDA was defined by the following criteria: *(i)* respiratory set-back with supplemental oxygen requirement of more than 30% and/or mechanical ventilation and/or *(ii)* left atrium to aortic root ratio of at least 1.4 in the echocardiogram, (iii) ductal size (>1.5 mm) and flow pattern, and/or (iv) decreased end-diastolic flow in the anterior cerebral artery with a resistance index of at least 0.85 based on cerebral ultrasound. Therapy was initiated according to a previously published algorithm [13]. When the PDA persisted despite pharmacological treatment and infants could not be weaned from mechanical ventilation, surgical closure with a vascular clip or suture was performed via a posterolateral thoracotomy.

Enrolled infants were classified into two groups according to treatment: *(i)* no PDA ligation (pharmacological closure with intravenous indomethacin or intravenous or oral ibuprofen, *N* = 74), and *(ii)* surgical closure by PDA ligation (*N* = 40). All parents provided written informed consent before LFT, and the Institutional Data Safety Committee approved this study.

### Lung function testing

LFT was performed in clinically stable children who had no lower airway infections in the preceding 3 weeks. The departmental protocol for LTF has previously been published [14]. Briefly, prior to testing, body weight and body length (crown to heel) were measured. At 15–30 min before LFT, sleep was induced by oral administration of chloral hydrate (50 mg∙kg^−1^). Sleeping infants were measured while in a supine position, with the neck supported by a neck roll in a neutral position. A compliant silicon infant mask (size 1, 2 or 3 as appropriate; Vital Signs Inc., Totowa, NJ, USA) was tightly placed over the nose and mouth.

Tidal breathing parameters (tidal volume [V_T_], respiratory rate [RR], minute ventilation [V’_E_], peak tidal inspiratory flow [PTIF], peak tidal expiratory flow [PTEF], and the ratio of time to peak tidal expiratory flow to expiratory time [t_ptef_/t_e_]) were initially measured by the deadspace free flow-through technique using custom-made equipment as previously described [15]. This technique allows long-term measurements in preterm infants because the face mask and flow sensor are continuously and thoroughly rinsed by a constant background flow which virtually eliminates dead space in the apparatus. The flow-through technique also allows continuous monitoring of mask leaks. After these initial measurements, respiratory lung mechanics (respiratory compliance [C_rs_] and respiratory resistance [R_rs_]) were measured by the occlusion technique using a balloon shutter. These measurements were performed using the MasterScreen™ BabyBody Plethysmograph (CareFusion, Höchberg, Germany), which also provides measurements of respiratory mechanics, whole-body plethysmography, and forced expiratory flow. Airway resistance (R_aw_) and functional residual capacity (FRC_pleth_) were also measured using the constant-volume baby body plethysmograph. The maximal expiratory flow at functional residual capacity (V’max_FRC_) was measured using the rapid thoraco-abdominal compression technique with the same equipment according to international guidelines [16].

Finally a multiple breath washout (MBW) technique was performed, with 4% sulfur hexafluoride (SF6) as a tracer gas, using the EXHALYZER D (EcoMedics AG, Duernten, Switzerland) to determine the lung volume involved in gas exchange (FRC_SF6_) and the lung clearance index (LCI) as a measure of ventilation inhomogeneity. Use of the same equipment in combination with the fast chemiluminescence NO-analyzer CLD 88 (EcoMedics AG, Duernten, Switzerland) allowed measurement of exhaled nitric oxide concentration (FeNO) and NO production (V’NO).

All flow and volume values were related to the body weight on the day of measurement to reduce the inter-subject variability. V’max_FRC_ was also expressed in standard deviation scores (Z-scores) that were based on sex, corrected age, and height-specific reference values of healthy infants published by Hoo et al. [17] and adjusted for the MasterScreen™ BabyBody by Lum et al. [18].

An arterialized capillary blood gas sample was taken at the end of LFT and analyzed using an ABL800 FLEX (Radiometer, Denmark). Heart rate and oxygen saturation were monitored continuously by a pulse oximeter (N-200; Nellcor, Hayward, California, USA) during the LFT. Complete data were obtained from almost all of the 114 infants regarding patient characteristics, occlusion test results, whole-body plethysmography, and blood gas analysis. The equipment for tidal breathing measurements was not available in 7 (6%) cases due to unplanned maintenance work. The rapid thoraco-abdominal compression technique for measurement of V’maxFRC (*N* = 95) and the SF_6_ MBW for measurement of FRC_SF6_ and LCI (*N* = 76) were added later in our lung function laboratory. The last added device (smallest number of measurements) was the Analyzer CLD 88 for measurement of FeNO (*N* = 45).

### Statistical methods

Patient characteristics and lung function parameters are given as rates or medians and interquartile ranges (IQRs). Data of infants with and without PDA ligation were compared by Fisher’s exact test, the Mann-Whitney rank test, or the Kruskal-Wallis rank test as appropriate. Spearman rank correlation was used to determine the correlation of the time of ligation with lung function parameters. A multivariate analysis of variance (MANCOVA) was used to investigate the effect of patient characteristics at birth and the day of measurements on various respiratory parameters. Statistical analysis was performed using Statgraphics Centurion® software (Version 16.0, Statpoint Inc., Herndon, Virginia, USA) and MedCalc (Version 9.2.0.2; MedCalc Software, Mariakerke, Belgium). A *p*-value less than 0.05 was considered statistically significant.

## Results

### Subjects

Table 1 shows the characteristics of the 114 enrolled VLBW infants who had PDA. Seventy-four (65%) infants received pharmacological treatment alone, and 40 (35%) infants received PDA ligation after failure of the initial pharmacological treatment. Infants in the PDA ligation group had a significantly lower gestational age (*p* < 0.01), lower birth weight (*p* < 0.018), and a higher incidence and a longer duration of mechanical ventilation (*p* < 0.001 for both). Moreover, assessment of risk-adjusted mortality (CRIB score) showed that infants in the PDA ligation group had higher CRIB scores (*p* = 0.003). The proportion of ELBW infants was the same in both groups (data not shown). There were also no statistically significant differences between the groups with respect to gender, fetal lung maturation, or surfactant treatment. Among the 40 (35%) infants treated with surgical ligation, the median (IQR) age at the time of surgery was 19 days.

**Table 1:** Characteristics of the 114 infants with PDA who were treated with and without PDA ligation

	Without PDA ligation	With PDA ligation	*p*-value
	*N* = 74	*N* = 40	
Neonatal period			
Male	29 (50%)	21 (53%)	0.171
Gestational age (weeks)	26 (25–28)	25 (24–26)	**<0.001**
Birth weight (g)	835 (690–990)	744 (630–860)	**0.018**
Birth weight <1000 g	58 (78%)	36 (90%)	0.120
Prenatal steroids^a^	55/72 (76%)	31/38 (82%)	0.531
Surfactant administration^a^	66/72 (92%)	36/39 (92%)	0.906
Duration of hsPDA (days)	10.5 (7 – 23)	19 (16 – 26)	**<0.001**
Mechanical ventilation >24 h	53 (72%)	40 (100%)	**<0.001**
Duration of mechanical ventilation (days)	14 (5 – 26)	26.5 (14.5 – 33.5)	**<0.001**
Duration of mechanical ventilation up to PDA closure (days)	6 (3 – 10)	12.5 (5.5 – 17.5)	**0.003**
CRIB score^a^	5 (1–8)	9 (5–11)	**0.003**
At day of lung function testing			
Age (days)	157 (140 – 176)	160.5 (137 – 186)	0.587
Postconceptional age (weeks)	48.3 (45.3 – 51.6)	48.0 (45.4 – 51.0)	0.622
Body weight (g)	4300 (3800 – 5100)	4422 (3795 – 5100)	0.478
Body length (cm)	54.5 (52.5 – 57.0)	55.3 (52.75 – 58.0)	0.448

Data represent median (interquartile range) or *n* (%), statistically significant *p*-values are printed in bold

^a^Total number is reduced because some data of outpatients were incomplete

At the day of LFT, there were no statistically significant differences between the groups regarding chronological and postmenstrual age, body weight, and body length. The LFT was performed at a median (IQR) postmenstrual age of 48.3 (45.3–51.6) weeks. None of the enrolled infants required additional oxygen or any respiratory support on the day of testing.

### Lung function testing

**Fig. 1:**
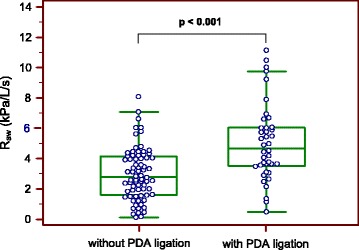
Airway resistance (R_aw_), measured by baby body plethysmography, of infants treated with and without PDA ligation

Table 2 shows the LFT results of infants in the 2 groups. Analysis of tidal breathing parameters indicated that the PDA ligation group had a lower V_T_ (*p* < 0.007) and a trend for lower PTEF (*p* = 0.078). Comparison of forced expiratory flow parameters indicated the ligation group had significantly lower V’max_FRC_ (*p* = 0.019) and corresponding z-score (*p* = 0.006). The respiratory mechanics were significantly different between both patient groups. Infants with PDA ligation had a significantly reduced compliance, increased airway resistance (Fig. 1) (*p* < 0.001 for both), and showed a trend for greater respiratory resistance (*p* = 0.076). Both patient groups had similar FRC_Pleth_, FRC_SF6_, and LCI as well as similar flow and concentration of exhaled NO, however, the PDA ligation group had significantly lower paO_2_ and higher paCO_2_ (*p* < 0.001 for both).

**Table 2:** Lung function testing of infants treated with and without PDA ligation

	*N*	hsPDA successfully treated with COX inhibitors	PDA ligation after unsuccessful treatment with COX inhibitors	*p*-value
Tidal breathing				
V_T_ (mL/kg)	107	5.96 (5.27 – 6.39)	5.15 (4.35 – 6.10)	**0.007**
RR (1/min)	107	40.0 (36.0 – 47.0)	43.5 (36.0 – 47.0)	0.132
V’_E_ (mL/min/kg)	107	234.2 (207.3 – 274.3)	221.6 (186.6 – 252.0)	0.137
PTIF (L/min/kg)	107	0.85 (0.77 – 1.0)	0.87 (0.71 – 0.98)	0.984
PTEF (L/min/kg)	107	0.77 (0.64 – 0.96)	0.71 (0.58 – 0.88)	0.078
t_ptef_/t_e_ (%)	107	18.1 (14.1 – 24.5)	17.2 (15.4 – 23.3)	0.896
Forced expiratory flow				
V’max _FRC_ (mL/s)	95	42.0 (25.0 – 61.0)	31.0 (13.0 – 52.0)	**0.019**
Z-Score V’max_FRC_	95	−1.82 (−2.48 – −1.07)	−2.53 (−3.0 – −1.74)	**0.006**
Respiratory Mechanics				
C_rs_ (mL/kPa/kg)	114	9.6 (8.1 – 10.6)	7.2 (6.3 – 9.1)	**<0.001**
R_rs_ (kPa/L/s)	114	6.6 (5.2 – 8.3)	7.5 (6.3 – 8.3)	0.076
R_aw_ (kPa/L/s)	114	2.84 (1.70 – 4.19)	4.81 (3.64 – 6.14)	**<0.001**
Lung volumes and lung clearing index				
FRC_Pleth_ (mL/kg)	114	20.9 (18.8 – 24.2)	21.1 (18.0 – 23.9)	0.852
FRC_SF6_ (mL/kg)	76	19.7 (15.6 – 24.3)	22.4 (18.6 – 25.2)	0.168
LCI	76	6.0 (5.5 – 6.8)	6.0 (5.7 – 6.9)	0.601
Exhalation gas analysis				
eNO (ppb)	45	9.0 (3.3 – 18.2)	7.9 (3.7 – 11.3)	0.851
V’_NO_ (nL/s)	45	11.8 (3.8 – 26.4)	14.4 (7.3 – 26.4)	0.617
Blood gas analysis				
paO_2_ (mmHg)	113	70.6 (60.7 – 80.7)	59.7 (50.8 – 70.9)	**<0.001**
paCO_2_ (mmHg)	113	40.9 (38.1 – 44.3)	45.1 (42.0 – 52.2)	**<0.001**

*N*: number of lung function tests, presented as median and (IQR), statistically significant *p*-values are printed in bold

### Multivariate analysis of variance

We used multivariate analysis of variance (MANCOVA) to investigate the impact of patient characteristics at birth and on the day of measurements as well as the durations of mechanical ventilation and hsPDA on maximal expiratory flow at functional residual capacity (V’max_FRC_), respiratory compliance (C_rs_)_,_ and airway resistance (R_aw_). The results indicate that the differences of V’max_FRC_ and C_rs_ between both patient groups mainly depended on patient characteristics (*p* < 0.05 for both), but R_aw_ did not. Moreover, PDA ligation had a significant effect on R_aw_ (*p* < 0.001), but not V’max_FRC_ and C_rs_.

## Discussion

There is an ongoing debate on the potential harms and benefits of PDA treatment in neonates. An hsPDA is thought to negatively affect pulmonary outcomes, however, the different treatment options for PDA (pharmacological, surgical, interventional) themselves exhibit potential risks and unclear benefits [1, 2, 19]. Chen et al. recently provided important data showing that preterm infants who received conservative management for PDA had a higher percentage of BPD than the infants without PDA, but LFT results were comparable between these two groups at discharge [20]. However, these authors did not investigate the effects of specific pharmacological or surgical PDA treatment on LFT. We herein retrospectively analyzed LFT data of a large cohort of VLBW infants at 48 postconceptional weeks after successful pharmacological PDA treatment or PDA ligation secondary to failed pharmacological treatment.

Our data shows that infants who underwent PDA ligation exhibited alterations in their lung function parameters as compared to those who received successful pharmacological treatment. Specifically, the PDA ligation group had reduced respiratory compliance, lower V’max_FRC_, increased airway resistance (R_aw_), tidal volume, forced expiratory flow, and impaired blood gases.

One major limitation of our study lies in the strong differences between the two groups: At birth the infants who received PDA ligation were more immature and sicker (with significantly higher CRIB scores), and had a longer duration of mechanical ventilation. These factors are associated with higher failure rates of pharmacological intervention for PDA in infants [21]. This is a common problem in the literature on PDA treatment, and due to a lack of randomized trials encompassing a non-treatment arm, researchers have to rely on retrospective analysis which impairs the possibility to establish causal relationships [1, 21]. In order to somewhat circumvent this problem, we conducted a multivariate analysis accounting for the differences in birth weight, gestational age, and durations of hsPDA and mechanical ventilation. This analysis showed that PDA ligation itself was an independent risk factor for increased airway resistance while all other parameters were not independently affected by PDA ligation.

After PDA ligation, we observed an increased airway resistance indicating upper airway obstruction that was independent of immaturity and postnatal development including durations of hsPDA and mechanical ventilation. Although patient characteristics influence the reduction of V’max_FRC_ and C_rs_, only PDA ligation seems to increase R_aw_ (Table 3). This increase is only weakly reflected by R_rs_, because R_aw_ (measured by whole-body plethysmography) primarily describes air conductivity of the upper airways, whereas R_rs_ (measured by an airway occlusion test) describes the resistive properties of the whole respiratory system, including the resistance of lung tissue. The increase of R_aw_ and the expiratory flow limitation during tidal breathing in infants who underwent PDA ligation points to problems in their upper airways. It is tempting to speculate that this proximal airway obstruction was due to unilateral vocal fold paralysis (UVFP) following injury of the recurrent laryngeal nerve. A recent meta-analysis indicated that UVFP occurred in up to 40% of children after surgical PDA closure [22]. In corroboration, adults with UVFP have increased R_aw_ and, accordingly decreased specific airway conductance [23]. Unfortunately, a laryngoscopic evaluation of vocal fold motility was only performed in a small number of infants in the present study, and this prevented statistical evaluation. In light of a possible increased risk of UVFP, it seems prudent to routinely perform laryngoscopic evaluation in VLBW infants after PDA ligation. LFT is an additional tool to identify infants with upper airway obstruction and to quantify the severity of the airflow impediment. Of note, LFT in infants is non-invasive, as opposed to laryngotracheoscopy, and might help detect incipient upper airway obstruction before the onset of clinical symptoms such as stridor or dyspnea.

**Table 3:** Multivariate analysis of variance (MANCOVA) of forced maximal flow (V’max_FRC_), respiratory compliance (C_rs_), and airway resistance (R_aw_) with patient characteristics as covariates

	V’max_FRC_		Respiratory compliance		Airway resistance	
Source	*F* ratio	*P* value	*F* ratio	*P* value	*F* ratio	*P* value
Covariates						
Gestational age	<0.01	0.945	0.19	0.667	0.02	0.885
Birth weight	4.99	**0.028**	2.98	0.087	<0.01	0.991
Postconceptional age	4.22	**0.043**	7.69	**0.007**	0.37	0.544
Body weight	2.44	**0.122**	6.48	**0.012**	1.11	0.295
Duration hsPDA	0.26	0.609	0.05	0.821	0.65	0.423
Duration mechanical ventilation	0.43	0.513	2.29	0.133	0.08	0.773
Main effect						
PDA ligation	2.73	0.102	1.68	0.198	22.00	**<0.001**

*F* ratios are based on the residual mean square error and *p*-values indicate the statistical significance of each factor

V’max_FRC_ (measured by the rapid thoracoabdominal compression technique) describes small airway conductivity and is highly dependent on maturity. Previous studies showed that V’max_FRC_ was lower in preterm infants, even in the absence of neonatal disease or therapy [24, 25]. Of note, the differences in tidal breathing parameters were smaller than in other LFT parameters; the only difference was a reduced V_T_ relative to body weight in the PDA ligation group. There were no significant differences in PTEF and respiratory rate, probably due to the high inter-subject variability of tidal breathing at this age [26–28] Also, the two groups had no significant differences in lung volume relative to body weight, lung clearance index, or in an inflammatory marker (exhaled FeNO).

In general, data on LFT in infants after PDA treatment, especially after ligation, is sparse. Only a few studies assessed the short-term effects of PDA ligation on pulmonary function in ventilated patients, and the results were contradictory. Two early case series from the late 1970s showed increased pulmonary compliance after surgical PDA closure in ventilated preterm infants [10, 11], and a more recent prospective study of 16 ventilated VLBW infants demonstrated improvements of dynamic compliance (C_dyn_), V_T_, and V’_E_ after PDA ligation, although R_aw_, mean airway pressure, and peak inspiratory pressure remained unchanged [12]. In contrast, McCurnin et al. [8] investigated the effect of PDA ligation in a premature baboon model and found no significant differences in C_dyn_, oxygenation index, or ventilation index (VI), but a trend towards higher Cdyn and lower VI in ligated animals. The *post mortem* static lung compliance was similar in both groups of baboons. Another study by the same group confirmed the modest effects of ligation on lung function parameters, but reported no improvement in alveolar surface area [9].

Our data shows that, compared to infants after successful pharmacological PDA treatment, patients after PDA ligation exhibited an increase in upper airway resistance and a decreased respiratory compliance at a postconceptional age of 48 weeks. The increased upper airway resistance seems to be associated with PDA surgery itself. In contrast, all the other impairments in pulmonary mechanics observed in ligated infants were due to the patient characteristics, and, despite all the heterogeneity between groups, several LFT parameters showed no significant differences. The differences in the LFT parameters were also reflected by blood gas analysis. We used arterialized capillary rather than arterial blood gas analysis because it is less invasive, easier to perform, and shows a good correlation with arterial blood gas analysis for most parameters.

There is an ongoing controversy regarding the indications and protocols for treatment of PDA [1, 2, 21, 29]. Herein, we provide data that might support an adverse association between some LFT parameters and PDA ligation. It is nonetheless unclear if this is a causal relationship or just an epiphenomenon due to the more severe illness of infants who required surgery. A recent study by Schena et al. found a higher incidence of BPD in infants after PDA ligation [30], but their multivariate analysis showed that a longer duration of hsPDA -- not surgery itself -- was an independent risk factor for BPD. In addition, there are contradictory results on the effects of pharmacological PDA treatment on BPD rates [29]. Furthermore, hsPDA and its treatment influences parameters that affect pulmonary outcome, but cannot be assessed by LFT, such as the pulmonary vasculature. Prospective trials that include a non-treatment arm are needed to address these issues.

## Conclusions

In conclusion, despite the retrospective nature of our analysis and the aforementioned differences between the two groups, we provide the first large cohort descriptive data on postnatal LFT results in patients treated for hsPDA using a panel of various techniques that were performed by a single experienced investigator. We suggest that LFT after discharge might help identify infants with increased risk for upper airway obstructions. Despite the potential harms, ligation provides definite PDA closure in infants after failed pharmacological treatment. Nonetheless, the optimal indication and time point for treatment are yet to be determined, and a careful follow-up of this high-risk population is mandatory in order to identify respiratory and other sequelae after PDA ligation.

## Abbreviations

BPD: Bronchopulmonary dysplasia
C_dyn_: Dynamic compliance
CRIB: Clinical risk index for babies
C_rs_: Respiratory compliance
ELBW: Extremely low birth weight (<1000 g)
FeNO: Fractional exhaled nitric oxide
FRC_Pleth_: Functional residual capacity measured by body plethysmography
FRC_SF6_: Functional residual capacity measured by SF_6_ multiple breath washout
hsPDA: Hemodynamically significant PDA
IMV: Intermittent mandatory ventilation
LCI: Lung clearance index
LFT: Lung function testing
MBW: Multiple-breath inert gas washout
MV: Mechanical ventilation
NLD: Neonatal lung disease
PDA: Patent ductus arteriosus
P_et_CO_2_: End-expiratory CO_2_ partial pressure
PNT: Pneumotachograph
PTEF: Peak tidal expiratory flow
PTIF: Peak tidal inspiratory flow
R_aw_: Airway resistance
RR: Respiratory rate
R_rs_: Respiratory resistance
SF_6_: Sulfur hexafluoride
TB: Tidal breathing
t_PTEF_/t_E_: Time to peak tidal expiratory flow related to the expiratory time
UVFP: Unilateral vocal fold paralysis
V’_E_: Minute ventilation
V’_max FRC_: Maximal expiratory flow at functional residual capacity
VI: Ventilation index
VLBW: Very low birth weight (<1500 g)
V_T_: Tidal volume
